# Adverse events associated with anti-EGFR therapies for the treatment of metastatic colorectal cancer

**DOI:** 10.3747/co.v17is1.615

**Published:** 2010-07

**Authors:** M. Fakih, M. Vincent

**Affiliations:** * Department of Medicine, Roswell Park Cancer Institute, Buffalo, New York; † London Health Sciences Centre, London, Ontario

**Keywords:** epidermal growth factor receptor, skin toxicity, cetuximab, panitumumab

## Abstract

The epidermal growth factor receptor (EGFR), a member of the ErbB family of receptor tyrosine kinases, plays an important role in the control of cell growth and differentiation. Disruption of its signaling leads to neoplastic cell proliferation, migration, stromal invasion, resistance to apoptosis, and angiogenesis.

EGFR is overexpressed in a variety of solid tumors, including colorectal cancer (CRC), and its overexpression is associated with poorer prognosis. One class of agents that is currently used to target EGFR in the treatment of metastatic CRC (mCRC) is the monoclonal antibodies. While the monoclonal antibody EGFR inhibitors lack many of the severe side effects commonly observed with cytotoxic chemotherapy, they are associated with a set of unique dermatological toxicities. This paper reviews the safety profile of the anti-EGFR monoclonal antibodies cetuximab and panitumumab in the treatment of mCRC.

## 1. INTRODUCTION

The epidermal growth factor receptor (EGFR), a member of the ErbB family of receptor tyrosine kinases^1^,^2^,^3^, is a transmembrane glycoprotein composed of an extracellular ligand-binding domain, a transmembrane lipophilic segment, and an intracellular protein kinase domain^4^.

EGFR is activated by EGF-like ligands, including EGF, transforming growth factor alpha (TGF-α), amphiregulin, heparin-binding EGF-like growth factor, betacellulin, and epiregulin^5^. Binding of these ligands results in EGFR dimerization, which leads to high-affinity ligand binding, activation of the intrinsic protein tyrosine kinase (TK) activity, and tyrosine autophosphorylation. Activation of the intracellular protein TK leads to recruitment and phosphorylation of several intracellular substrates, triggering a variety of cellular responses including cell division, survival, motility, invasion, adhesion, and cellular repair^6^,^7^. EGFR therefore plays an important role in the control of cell growth and differentiation^8^, and disruption of its signaling leads to neoplastic cell proliferation, migration, stromal invasion, resistance to apoptosis, and angiogenesis^9^.

EGFR is overexpressed in a variety of solid tumors, including colorectal cancer (CRC), squamous cell cancer of the head and neck, and non-small-cell lung cancer. EGFR overexpression is associated with poorer prognosis in these malignancies^10^–^14^ and, in CRC, may be associated with an advanced disease stage^15^–^18^.

One class of agents that is currently used to target EGFR in the treatment of metastatic CRC (mCRC) is the monoclonal antibodies (MoAbs), which compete with endogenous ligands, including EGF and TGF-α to block ligand-dependant activation of EGFR, and induce receptor internalization and consequent downregulation^19^. This paper reviews the safety profile of the anti-EGFR MoAbs cetuximab and panitumumab in the treatment of mCRC.

## 2. DERMATOLOGICAL TOXICITIES

While the MoAb EGFR inhibitors lack many of the severe side effects commonly observed with cytotoxic chemotherapy, they are associated with a set of unique dermatological toxicities. The majority of patients treated with a MoAb EGFR inhibitor will experience dermatological side effects, most notably the papulopustular skin rash, which can impact quality of life and affect adherence to therapy^20^.

EGFR is expressed in the basal layer of the epidermis and contributes to the stimulation of epidermal growth, inhibition of differentiation, and acceleration of wound healing. Inhibition of EGFR results in impaired growth and migration of keratinocytes, and inflammatory chemokine expression by these cells. The resulting inflammatory cell recruitment and subsequent cutaneous injury account for the majority of dermatological symptoms associated with anti-EGFR therapy including papulopustular eruption, hair growth disorders, periungual and nail plate abnormalities, xerosis, telangiectasias, and pruritus^13^,^21^–^29^. Disruptions to this barrier may also promote bacterial overgrowth^30^, further exacerbating injury to the cutaneous tissue.

Skin toxicities have been reported in 80–95% of patients with mCRC treated with cetuximab and panitumumab monotherapy (Tables 1, 2)^31^, with a similar frequency seen in trials of cetuximab in combination with chemotherapy (Table 3). The most severe skin toxicities (grades 3–4) have been seen in trials combining bevacizumab with the EGFR inhibitors^32^–^34^ In the CAIRO2 trial, the addition of cetuximab to capecitabine, oxaliplatin and bevacizumab in the first-line treatment of mCRC did not result in excessive toxicity^33^. However, an overall increase in grade 3–4 toxicity was seen in the cetuximab arm, which was fully attributed to cetuximab-related skin toxicity. In the BOND trial, grade 3–4 skin toxicity occurred at a rate of 13% in the group treated with cetuximab and irinotecan^13^. In BOND2, the grade 3 skin rash was observed in 21% of patients treated with a combination of cetuximab, irinotecan, and bevacizumab^35^, suggesting that there is considerable worsening of skin toxicity with the addition of bevacizumab to an anti-EGRF treatment regimen.

The PACCE (Panitumumab Advanced Colorectal Cancer Evaluation) study evaluated the efficacy and safety of adding panitumumab to combination chemotherapy with bevacizumab for the first-line treatment of mCRC^34^. PACCE was stopped early when a planned interim analysis revealed an increased incidence of toxicity with no improvement of increased efficacy in the panitumumab arm. Grade 3–4 skin toxicity was observed in 36% of patients treated with panitumumab.

### 2.1 Papulopustular rash

The most common adverse event among mCRC patients treated with the anti-EGFR MoAbs is the papulopustular rash, characterized by erythematous inter- and intrafollicular papulopustules and commonly affecting sun-exposed areas of the body^36^ such as the face, neck, shoulders, upper body, and scalp^37^,^38^. In clinical trials of cetuximab and panitumumab monotherapy, papulopustular rash occurred in the majority of patients, but in most cases were mild-to-moderate in severity (Tables 1, 2).

The onset of rash is early, generally developing over a period of six weeks after starting treatment^39^. The first week is characterized by sensory disturbance, erythema, and edema, followed by papulopustular eruption during the second week. Crusting appears by Week 4. If treated successfully, the papulopustular eruptions disappear and make way for erythema and dry skin by Week 6. In some cases, the rash may improve spontaneously^40^, but may also persist^26^. Phase I escalation studies have shown the rash to be dose-dependant^40^–^42^.

Although the rash may resemble acne vulgaris, it is clinically and histologically different. Therefore, terms that imply a similarity with acne, such as “acneiform rash”, “acneiform follicular rash”, “acne-like rash”, “maculopapular rash”, or “monomorphic pustular lesions”, should not be used to describe the papulopustular rash associated with anti-EGFR therapy^36^,^37^,^43^,^44^.

#### 2.1.1 Correlation between the efficacy of EGFR inhibitors and the occurrence of rash

Data from the clinical trials of cetuximab and panitumumab in the treatment of mCRC suggest a positive correlation between the presence and severity of rash and survival^13^,^32^,^45^–^49^. In the first phase II open-label trial of cetuximab monotherapy for the treatment of refractory mCRC, longer survival times were observed in patients who experienced a rash of any grade compared with patients who did not have a rash (*p* = 0.02)^32^. In the pivotal BOND study comparing cetuximab in combination with irinotecan with cetuximab alone for the treatment of mCRC, patients with skin reactions had higher response rates than patients without skin reaction (25.8% vs. 6.3% in the combination group; 13.0% vs. 0% in the monotherapy group; *p* = 0.005)^13^.

Similar results have been observed in phase II and III studies of panitumumab. In a phase II study of 148 patients with EGFR-positive mCRC, grades 2–4 skin toxicity was associated with longer PFS (HR 0.67; 95% CI 0.50 to 0.90) and OS (HR 0.72; 95% CI 0.54 to 0.97) compared with grades 0–1 skin toxicity^49^. In the pivotal phase III, open-label trial comparing panitumumab monotherapy with best supportive care for the treatment of mCRC, exploratory analysis revealed a trend toward longer progression-free survival (HR 0.62; 95% CI 0.44–0.88) and overall survival (HR 0.59, 95% CI 0.42–0.85) in patients with grade 2–4 skin toxicity compared with patients with grade 1 skin toxicity^50^.

The correlation between rash and response to the anti-EGFR treatment suggests that treatment response might be optimized by increasing the dose until the appearance of rash. The phase I/II EVEREST (Evaluation of Various Erbitux Regimens by Means of Skin and Tumor Biopsies) trial randomly assigned patients with no rash or grade I rash to treatment with standard-dose cetuximab (250 mg/m^2^/week) plus irinotecan or an increasing dose of cetuximab (50 mg/m^2^ every two weeks until grade 2 or higher toxicity, tumor response, to a maximum dose of 500 mg/m^2^)^51^. Skin toxicity and response rates both increased with dose escalation. Mean PFS was 4.8 months in the dose-escalation group compared with 3.9 months in those who received standard-dose cetuximab^51^. As KRAS mutation status has been shown to be a predictor of tumor response to anti-EGFR treatment, the EVEREST trial sought to determine whether dose escalation would also be able to induce a response in patients with KRAS mutations. KRAS and skin toxicity were found to be independent predictors of outcomes. Among patients with wild-type KRAS tumors and grade 0–1 rash, dose escalation improved response rates compared with the standard-dose group (46.4% vs. 21.1%). However, none of the patients with KRAS mutations achieved a response, regardless of the dose^51^.

These results suggest that the characteristic rash associated with EGFR inhibitors may have potential as a surrogate marker of efficacy in patients with KRAS wild type tumors.

#### 2.1.2 Management of skin rash associated with cetuximab and panitumumab

**Figure 1:**
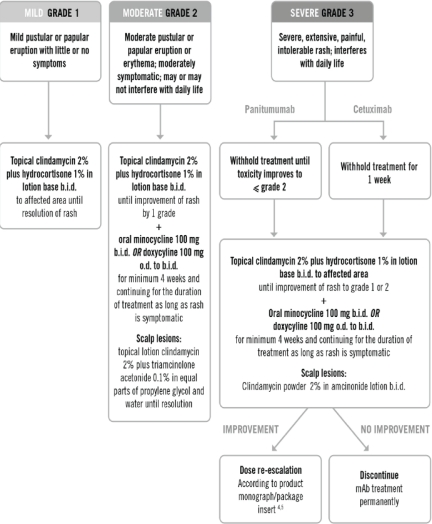
*Treatment recommendations for rash mediated by monoclonal antibody targeting of epidermal growth factor receptor, by severity (*Adapted from the BC Cancer Agency’s EGFR inhibitors rash protocol.*)*

Canadian guidelines have recently been developed for the prevention and management of dermatological toxicities associated with anti-EGFR MoAb treatment^52^. General principles include practicing sun-protective measures and avoidance of activities and products that are likely to dry the skin (e.g. long, hot showers; alcohol-based/perfumed products; over-the-counter acne medications). Oatmeal baths and creams may provide symptomatic relief. Management should be individualized according to the type, severity, and location of the rash. Specific treatment recommendations for mild, moderate, and severe rash are outlined in the British Columbia Cancer Agency’s rash protocol for EGFR inhibitors (Fig. 1). Twice daily application of topical clindamycin 2% plus hydrocortisone 1% in a lotion base is recommended for the treatment of mild rash. Moderate and severe rash may require the addition of oral minocycline or doxycycline.

Researchers at the Memorial Sloan-Kettering Cancer Center evaluated the ability of topical tazarotene, with or without oral minocycline, to reduce or prevent papulopustular rash when administered in conjunction with cetuximab therapy^53^. Forty-eight patients with mCRC who were about to start therapy with cetuximab were randomly assigned to receive daily treatment with oral minocycline (n = 24) or placebo (n = 24), and topical tazarotene on either the left or right side of their face. Both therapies were administered for eight weeks. During the first four weeks, minocycline treatment was associated with significantly fewer facial lesions and lower rates of severe itch compared with placebo (20% vs 50%, p = 0.05). By Week 8, these differences were no longer significant. Tazarotene application did not produce any clinical benefit and was, in fact, associated with significant irritation, resulting in its discontinuation in one-third of patients.

The largest randomized prospective study to demonstrate the efficacy of prophylactic intervention in reducing the risk of skin toxicity with an anti-EGFR therapy was the STEPP (Skin Toxicity Evaluation Protocol with Panitumumab) trial. The STEPP trial evaluated the differences between pre-emptive and reactive treatments for skin toxicities associated with EGFR inhibition by panitumumab in 58 patients receiving panitumumab plus FOLFIRI or irinotecan-only chemotherapy for second-line treatment of mCRC^54^. Patients with mCRC who had previously failed treatment with oxaliplatin-based chemotherapy were randomly assigned to biweekly treatment with FOLFIRI–based chemotherapy plus panitumumab or treatment with irinotecan-based chemotherapy plus panitumumab every three weeks. Within each treatment group, patients were randomly assigned to receive skin toxicity treatment 24 hours before the first panitumumab dose and then daily through Week 6 (preemptive) or after skin toxicity developed (reactive)^54^. Skin toxicity treatment included the use of skin moisturizers, sunscreen, 1% hydrocortisone cream, and doxycycline 100 mg bid. Preemptive treatment reduced the incidence of grade 2 or greater skin toxicities by more than 50% compared with reactive treatment, without additional side effects. Time to severe skin toxicity and time to first occurrence of a grade 2 or greater skin toxicity were also significantly delayed by preemptive treatment. Prophylactic management of skin toxicity was not associated with any reduction in efficacy when compared to the reactive skin toxicity arm.

In addition to treatment with topical and oral antibiotics, specific dose reductions and treatment delays are recommended for patients who develop severe rash on cetuximab (Table 4)^55^.

### 2.2 Xerosis

Xerosis, or excessive dryness of the skin, is generally characterized by diffuse, fine scaling. Xerosis occurs in up to 35% of patients treated with an EGFR inhibitor^56^, and is more common among older patients or those with a history of atopic eczema^37^. Xerosis may be complicated by chronic asteototic eczema, or “winter eczema”, which is characterized by pruritic, dry, cracked, and polygonally fissured skin with irregular scaling.

Xerosis is associated with a significant decrease in free fatty acids in the stratum corneum. Cutaneous loss of these fatty acids increases transepidermal water loss, causing the cells to shrink and reducing the skin’s elasticity. This disruption of the epidermal layer can lead to inflammation and an increased risk of infection by *Staphylococcus aureus* or, less commonly, herpes simplex virus type I^23^,^24^. Systemic and/or topical antibiotics may be required^57^. Pruritus may be alleviated with antihistamines^39^, and anecdotal reports suggest that pregabalin may control pruritus associated with cetuximab therapy^58^.

Painful fissures may appear on the palms, fingertips, soles of the feet and toes, and on the lips^20^,^21^,^24^,^59^,^60^. Fissures should be treated with emollients, and sealed with cyanoacrylate or flurandernolide tape that delivers high-potency steroids and protects against mechanical trauma^61^.

### 2.3 Paronychia

Paronychia is an infection that occurs where the nail and skin meet at the side or the base of a fingernail or toenail. Occurring in 10% to 15% of patients treated with cetuximab and gefitinib^24^,^26^,^29^,^62^, this side effect generally presents four to eight weeks from the start of treatment with an EGFR inhibitor MoAb. Paronychia associated with EGFR blockade is characterized by an erythematous and painful inflammation of the nail fold, which may swell and form granulation tissue^37^. The nails may become brittle and slower growing. In severe cases, paronychia perungual abscess and pyogenic granuloma of the nail fold may develop^62^. Secondary bacterial (*S. aureus*) or fungal (*Candida albicans*) infection is common in paronychia associated with EGFR inhibition^29^,^63^,^64^.

Minocycline or doxycycline 100 mg bid and high-potency topical steroids may be effective in the treatment of paronychia^61^. Extreme cases may require nail local steroid injections or nail fulguration.

### 2.4 Hair changes

Some patients who undergo treatment with an EGFR inhibitor may experience changes to their hair, most notably an increased growth of the eyelashes (trichomegaly)^37^,^65^,^66^. However, the patient’s scalp hair may become finer, brittle, and curly, and hypertrichosis of the face may develop^67^, suggesting that the mechanism regulating hair growth may differ in different parts of the body^66^. In three clinical studies investigating the safety and efficacy of panitumumab monotherapy in mCRC in a single institution, hirsutism was reported in half of women who received panitumumab for more than six weeks^68^.

Hair changes usually appear later during the course of treatment – two to five months after beginning treatment – and generally resolve within a month of discontinuing treatment^37^.

### 2.5 Telangiectasias and hyperpigmentation

Telangiectasias are small dilated blood vessels that develop in a small proportion of patients taking EGFR inhibitors, generally appearing on the face, chest, back, and limbs. Hyperpigmentation may result from fading telangiectasias^24^,^59^,^60^ or as a consequence of inflammation. Because telangiectasias and hyperpigmentation usually occur as a result of photosensitivity, patients being treated with an EGFR inhibitor should be counseled to practice sun protection. The Canadian Dermatology Association has outlined sun protection for the general population, which includes wearing an SPF 30 broad-spectrum sunscreen, scheduling outdoor activities before 11 am and after 4 pm, and wearing appropriate clothing to cover the skin, including hat. Darker-skinned individuals, in particular, are susceptible to hyperpigmentation^24^,^43^,^59^,^60^,^68^,^69^.

### 2.6 Radiation dermatitis

With the increasing use of EGFR inhibitors with or following radiotherapy, recent reports have indicated a potential for cetuximab to enhance the severity of radiation dermatitis^70^–^72^. The radiation oncology department of the University of Dusseldorf, Germany, observed two cases of unusually severe radiation dermatitis among a small group of five patients with head and neck cancer (HNC) treated with irradiation and concurrent cetuximab^71^. The appearance of these cases prompted the researchers to conduct a survey of members of the EORTC Head and Neck Radiation Oncology Group. Among 71 HNC patients from 15 institutions who had been treated with cetuximab and concurrent radiotherapy and for whom information on dermatological reactions was available, 15 and 20 patients developed grade 3 and grade 4 radiation dermatitis (49%), respectively^73^. In another recent report of 13 consecutive patients with HNC treated with concurrent cetuximab and radiotherapy, 10 (77%) experienced severe skin reactions (grade 3–4), which were associated with low treatment compliance and delays in completing RT^74^.

These results are in contradiction to results of a large, multinational, randomized study, where the concurrent addition of cetuximab to radiation treatment did not increase the rate of grade 3–4 radiation dermatitis (11% cetuximab plus radiotherapy vs 8.5% radiotherapy alone; *p* = 0.27)^75^. A potential reason for this discrepancy in the German study is selection bias, as institutions that had observed cases of severe radiation dermatitis may have been more likely to respond to the survey than those who had not. As well, the total number of patients might have been underestimated due to the lack of formal registry of cetuximab patients in most institutions. Another potential reason for the increase in radiation dermatitis observed in the more recent studies is the removal of radiation dermatitis as a dose-limiting toxicity of radiotherapy, since the introduction of megavoltage radiotherapy. As a result, severe radiation dermatitis may have been underreported in the earlier trial^71^,^73^.

## 3. OCULAR TOXICITIES

Ocular toxicities such as conjunctivitis and blepharitis with increased lacrimation have been reported for both cetuximab and panitumumab^47^,^76^.

Blepharitis, or inflammation of the lid margin, results from inflammation of the meibomian glands, which contain EGFR-expressing cells. Symptoms include itching and watering of the eyes and lids, and crusting of the lashes. In general, treatment of blepharitis includes warm compresses, eyelid scrubs, and topical antibiotic. Eyelid cultures should be obtained if the condition fails to improve with these measures^77^. While there are no clear guidelines for dose-modifications with EGFR inhibitors, in the case of cetuximab, Dranko et al. recommend following the dose-modification regimen recommended for papulomacular rash (Table 5)^77^,^78^.

## 4. HYPOMAGNESEMIA

Hypomagnesemia has emerged as a relatively common side effect of cetuximab and panitumumab therapy^48^,^79^–^84^. In the early trials of anti-EGFR therapies, the incidence of hypomagnesemia was underestimated^80^,^81^, likely because these trials focused on patients with overt hypomagnesemia and who therefore had time to develop hypomagnesemia during the relatively short treatment intervals^85^–^87^. However, postmarketing experience with the anti-EGFR MoAbs began to reveal reports of severe hypomagnesemia^88^, and the incidence was found to increase with increasing duration of treatment^87^. In a retrospective review of 114 mCRC patients treated with cetuximab at the Roswell Park Cancer Center in Buffalo, grade 3–4 hypomagnesemia was observed in 5%, 23%, and 47% of patients who were treated with cetuximab for less than three months, for three to six months, and for more than six months, respectively^87^.

Panitumumab is associated with a similar risk of hypomagnesemia. Among patients with mCRC who were treated with panitumumab, with or without best supportive care, magnesium concentrations were reduced in 36% of the 231 patients treated with panitumumab (3% grade 3–4) compared with 1% of those receiving best supportive care alone^50^. A lower frequency of hypomagnesemia was observed in the PACCE trial, where the combination of panitumumab, fluorouracil, oxaliplatin, and leucovorin in the first-line treatment of mCRC was associated with only a 4% incidence of grade 3–4 hypomagnesemia^34^. This low frequency of hypomagnesemia has been attributed to a lack of stringent guidelines for magnesium monitoring^84^.

The timing of the onset of hypomagnesemia during treatment with anti-EGFR MoAbs can be inferred from the rate of magnesium loss and the duration of treatment. Among 98 consecutive patients with mCRC treated with anti-EGFR MoAbs in a Belgium study, 97% experienced a progressive decrease in serum magnesium concentrations after initiation of treatment, with a median time to hypomagnesemia of 99 days (range 12–639 days)^79^. Serum magnesium concentrations returned to normal shortly after discontinuation of the EGFR inhibitor^79^. The slope of the change in serum magnesium concentrations from baseline was calculated with three early time points and was found to correlate well with the slope of the entire dataset, suggesting that early characterization of magnesium wasting might be possible in clinical practice.

The mechanisms responsible for hypomagnesemia in association with anti-EGFR MoAbs have not been well defined. Increased EGFR expression in the ascending loop of Henle, where 70% of filtered magnesium is reabsorbed, may result in damage to the renal tubule and interfere with magnesium transport^85^,^88^.

Symptoms of hypomagnesemia can be cardiovascular, neuromuscular, or behavioral^89^. Cardiovascular symptoms include ventricular ectopic beats, hypertension, enhancement of digoxin-induced dysrhythmia, and cardiomyopathies and, in more severe cases, ventricular tachycardia, ventricular fibrillation, atrial fibrillation, and multifocal atrial tachycardia. Neuromuscular and behavioral symptoms include weakness, confusion, tetany, agitation, tremors and depression and, in more severe cases, convulsions, psychosis, ataxia, spasticity, and delirium. Hypocalcemia has been reported in association with hypomagnesemia^87^ and can contribute to neuromuscular symptoms. This hypomagnesimic hypocalcemia can only be corrected by replacing magnesium levels. The pathophysiology of hypocalcemia in this setting is related to hypomagnesemiainduced PTH resistance.

The impact of hypomagnesemia in patients undergoing anti-EGFR treatment for mCRC has been underestimated, most likely because magnesium levels are rarely measured during routine screening^84^. Patients’ electrolytes should be periodically monitored during – and for eight weeks after – the completion of anti-EGFR therapy. Hypomagnesemia should be suspected in mCRC patients being treated with cetuximab or panitumumab who present with chronic diarrhea, hypocalcemia, refractory hypokalemia, and ventricular arrhythmia^84^.

Hypokalemia has similarly been associated with anti-EGFR therapy, although to a lesser extent than hypomagnesemia. The exact mechanism has not been elucidated and it has been observed in the absence of diarrhea. Hypokalemia typically responds well to oral potassium supplementation.

### 4.1 Management of hypomagnesemia

Management of hypomagnesemia is dependent on the grade of severity outlined in Table 6. Patients with grade I hypomagnesemia are generally asymptomatic and do not require replacement therapy^90^. In patients with grade 2 hypomagnesemia, oral supplementation is generally ineffective and poorly tolerated due to diarrhea^79^,^87^. Weekly intravenous treatment with magnesium sulfate 4 g has been shown to be effective for patients with magnesium levels of 0.9 to 1.0 mg/dL (0.37–0.41 mmol/L)^90^. For patients with grade 2 hypomagnesemia who are asymptomatic and without cardiac risk factors, weekly monitoring without magnesium supplementation may be considered.

Grade 3–4 hypomagnesemia is associated with symptoms of fatigue, cramps, and somnolence^79^, which are often attributed to cytotoxic chemotherapy and, therefore, go unreported. However, the patient’s energy level and performance status may be improved by normalizing magnesium levels of those with grade 3–4 hypomagnesemia^90^. Replacement therapy is particularly important for these patients, as grade 3–4 hypomagnesemia puts the patient at increased risk for cardiac arrhythmia, which may lead to sudden death^78^.

Management of grade 3–4 hypomagnesemia is challenging, requiring intravenous treatment with magnesium sulfate 6 to 10 g a minimum of two times per week. In severe cases, daily supplementation may be necessary, which can be extremely limiting and inconvenient for the patient^90^. In such cases, a four-to-eight-week break from EGFR inhibition may be considered, as magnesium concentrations return to normal approximately four to eight weeks after discontinuation. The patient may then be rechallenged with the EGFR inhibitor following reversal of the hypomagnesemia^90^.

## 5. DIARRHEA

Grade 3–4 diarrhea occurred in up to 2% of patients in the EGFR inhibitor monotherapy trials (Tables 1, 2). The incidence and severity of diarrhea is increased when cetuximab or panitumumab is given in combination with chemotherapy (Table 3), but is generally in the range expected for irinotecan therapy.

Grade 3–4 diarrhea occurred in up to 28% of patients in trials combining an EGFR inhibitor with chemotherapy (Table 3). In the CRYSTAL trial, the rate of grade 3–4 diarrhea was increased by the addition of cetuximab to FOLFIRI, compared with FOLFIRI alone (15.2% vs. 10.5%)^48^. In CAIRO2^33^, cetuximab increased the rate of grade 1–2 diarrhea, but not grade 3–4 diarrhea, when added to a regimen of capecitabine, oxaliplatin and bevacizumab.

The combination of panitumumab and IFL (5-fluorouracil [5-FU], leucovorin [LV], and irinotecan) is not recommended due to the high incidence of diarrhea seen in clinical trials. A phase II trial assessed the incidence of grade 3–4 diarrhea in mCRC patients treated with panitumumab in combination with first-line irinotecan-containing regimens^91^. Patients were initially treated with panitumumab (2.5 mg/kg weekly via a 1-hour infusion) in combination with IFL. However, the protocol was later amended to substitute folinic acid, 5-FU, and irinotecan (FOLFIRI) for IFL due to toxicity with IFL. In all, 19 patients were treated with IFL and 24 with FOLFIRI in combination with panitumumab. Grade 3–4 diarrhea was observed in 58% of patients in the IFL group and 25% of patients in the FOLFIRI group. In the PACCE trial^34^, the addition of panitumumab to either bevacizumab/irinotecan-based chemotherapy or to bevacizumab/oxaliplatin-based chemotherapy resulted in an increased incidence of grade 3–4 diarrhea, compared with the chemotherapy regimens alone.

Management of diarrhea should be aggressive, with treatment including loperamide or diphenoxylate^92^. General management may include bowel rest, hydration, and replacement of electrolytes. Hospitalization is required for patients with dehydration, fever, neutropenia, or nausea and vomiting that prevents adequate oral hydration^93^.

## 6. INFUSION REACTIONS

Severe infusion reactions have been reported in approximately 3.5–7.5% of mCRC patients treated with cetuximab (Table 1). In randomized trials of panitumumab monotherapy in mCRC, infusion-related reactions of all grade occurred in 0.6–3% of patients (Table 2).

A higher incidence of infusion reactions associated with cetuximab treatment was recently reported in a study in Tennessee and North Carolina, which included data for 88 patients in clinical trials and 55 patients outside of trials^94^. Among those in clinical trials, the grade 3–4 IRs occurred at a rate of 22%. However, rates of hypersensitivity reactions were much lower (< 1%) in most centers in the Northeast.

Most severe (grade 4) infusion reactions with cetuximab occur within a few minutes of taking the first dose. However, Needle et al. reported that 33% of grade 3–4 infusion reactions occurred after the second dose; less severe reactions may appear with subsequent treatments, suggesting differences in the underlying mechanisms responsible for mild and severe infusion reactions^95^.

Severe hypersensitivity reactions to cetuximab are thought to be largely due to IgE-mediated anaphylaxis, associated with the preexistence of IgE antibodies prior to treatment with cetuximab^96^. Following previous exposure to an antigen, IgE reaginic antibodies are released into the circulation by plasma cells derived from B lymphocytes under the influence of helper T-cells. These antibodies bind to receptors on tissue mast cells or blood-borne basophils, thereby sensitizing them. Subsequent reexposure to the antigen cross-links the Fab portions of two surface-bound IgE molecules, activating the cell and triggering the release of chemical mediators. Following reports of increased hypersensitivity reactions to cetuximab in the southeastern United States, IgE antibodies against cetuximab were detected in pretreatment blood samples of 68% of patients who had a hypersensitivity reaction to the drug. In contrast, IgE antibodies were detected in only 2% of those without a reaction (*p* < 0.001). The IgE antibody was discovered to be specific for the sugar galactose-α-1,3-galactose, expressed on the Fab portion of the cetuximab heavy chain. While the reason for the regional distribution of IgE antibodies to galactose-α-1,3-galactose in the United States is unclear, tick bites have been proposed as a potential etiology^96^.

The mechanism of panitumumab hypersensitivity reactions is not clearly understood. In a phase III trial of panitumumab monotherapy in patients with mCRC, 1.4% of patients tested positive for neutralizing antibodies^47^. However, a correlation between neutralizing antibodies and infusion reactions has yet to be demonstrated.

Patients may be able to continue treatment with an EGFR inhibitor following mild to moderate infusion reactions. Nielsen et al. described two patients with grade 2 infusion reactions to cetuximab, who were successfully rechallenged with cetuximab under controlled conditions^97^. Prednisone and antihistamines were administered prior to treatment, and the cetuximab infusion was started on a low rate, with gradual titration. The patients showed no evidence of an acute reaction during or after the cetuximab infusion and were able to continue treatment under the same treatment protocol. More recently, the addition of corticosteroids to antihistamines prior to treatment with cetuximab has been shown to reduce infusion-related reactions, without altering anti-tumor efficacy^98^.

There are also limited data demonstrating successful treatment with an alternative anti-EGFR MoAb following a severe infusion reaction with one anti-EGFR MoAb. Saif et al. described two mCRC patients with severe infusion reactions to panitumumab who were successfully challenged with cetuximab^99^, and three patients with severe hypersensitivity reactions to cetuximab who were successfully challenged with panitumumab^100^. The two patients who were switched to cetuximab received premedication with prednisone and antihistamines, and were treated with cetuximab according to a prolonged infusion time and gradual dose escalation^99^. None of the three patients successfully switched to panitumumab received pretreatment^100^.

## 7. CONCLUSIONS

The majority of patients treated with an MoAb EGFR inhibitor for mCRC will experience dermatological side effects, the most common of which is the papulopustular skin rash, which occurs early during the course of treatment and can impact the patient’s quality of life. The severity of the rash is dose-dependant and is also correlated with efficacy of treatment. Therefore, every effort should be made to ensure adherence to therapy. Most cases are mild-to-moderate in nature and will respond to treatment with topical antibiotics. More severe cases may require the addition of oral antibiotics, along with dose reduction or treatment delays. Preemptive treatment has been shown to delay the time to skin toxicity. Based on two randomized studies, the prophylactic use of systemic oral antibiotics, namely doxycycline or minocycline, reduces the risk of grade 2 and higher skin toxicity. While this practice may eliminate the reliability of skin toxicity as a predictive factor of response, it does not reduce anti-EGFR efficacy. Given the inconvenience associated with severe skin toxicities, many practices have moved to prophylactic oral antibiotic administration.

Other dermatological side effects of both cetuximab and panitumumab include xerosis, fissures, hyperpigmentation, and changes to the hair and nails. Recent reports have also indicated a potential for cetuximab to enhance the severity of radiation dermatitis.

Less common but important side effects include diarrhea and infusion reactions. Hypomagnesemia has emerged more recently as a side effect of EGFR inhibitors and should be considered in patients who develop fatigue and muscle weakness on therapy. Serum magnesium levels should be monitored routinely in patients undergoing treatment with cetuximab or panitumumab for mCRC.

## Figures and Tables

**Table I.** AEs in cetuximab monotherapy trials^31^

	*Saltz et al. (2004)*	*Cunningham et al. (2004)*	*Lenz et al. (2006)*	*Jonker et al. (2007)*	*Wierzbicki et al. (2008)*
*n* of patients	57	115	346	287	85
Any AE, *n* (%)	NR	50 (43.5)	NR	226 (78.5)	81 (95.3)
Any skin toxicity (%)	88	80	82.9	88.6	NR
Phase	II	II/III	II	III	II
Grade 3/4 AEs, %	Acne, 16; asthenia, 4; atrial fibrillation, 2; hypokalemia, 2; rash, 2; vomiting, 2; confusion, 2; diarrhea, 2; headache, 2	Dyspnea, 13.0; asthenia, 10.4; acne-like rash, 5.2; abdominal pain, 5.2; nausea/vomiting, 4.3; anemia, 2.6; diarrhea, 1.7; thrombocytopenia, 0.9; stomatitis, 0.9	Acne, 4.9; asthenia, 2.0; headache, 1.2; diarrhea, 1.2; nausea, 0.6; dry skin, 0.6; fever, 0.3	Fatigue, 33.0; dyspnea, 16.3; abdominal pain, 13.2; pain–other, 14.9; infection without neutropenia, 12.8: rash or desquamation, 11.8; hypomagnesemia, 5.8; edema, 5.2; anorexia, 8.3; constipation, 3.5; nausea, 5.6; vomiting, 5.6; confusion, 5.6	Dermatitis, 4.7; hypomagnesemia, 4.7; dyspnea 2.4; headache, 1.2
Onset of skin toxicity	1–3 wks	1–3 wks	8–19 days	NR	NR
Infusion reactions, type, *n* (%) *n* of patients	Allergic reactions, 3 (5)	Hypersensitivity reaction, 4 (3.5)	Hypersensitivity reaction, 26 (7.5)	Hypersensitivity reaction, 13 (4.5)	Infusion reaction grade ≥3, (3.5)

**Table II.** AEs in panitumumab monotherapy trials^31^

	*Van Cutsem et al. (2007)*	*Van Cutsem et al. (2008)*	*Berlin et al. (2006)*	*Hecht et al. (2008)*	*Hecht et al. (2007)*
	229	176	93	203	148
Any AE, *n* (%)	79 (35)	32 (18)	23 (25)	88 (42)	18 (12)
Any skin toxicity (%)	90%	NR	96	NR	95
Phase	III	II	II	II	II
Grade 3/4 AE, %	Acneiform rash, 7.4; abdominal pain, 7.4; erythema, 5.2; dyspnea, 4.8; fatigue, 4.4; anorexia, 3.5; asthenia, 3.1; constipation, 2.6; pruritus, 2.2; skin exfoliation, 2.2; vomiting, 2.2; hypomagnesemia, 3.0; back pain, 1.7; paronychia, 1.3; diarrhea, 1.3; nausea, 0.9; rash, 0.9; skin fissures, 0.9; edema, 0.9; cough 0.4	Acne, 6.2; erythema, 5.1; rash, 4.5; other skin manifestations, 2.3; paronychia, 1.7; pruritus, 1.1; skin exfoliation, 0.6; diarrhea, 0.6; conjunctivitis, 0.6	Acneiform rash, 9.9; erythema, 6.6; rash, 3.3; pruritus, 2.2; paronychia, 2.2; hypokalemia, 2.2; exfoliation, 1.1; skin fissures, 1.1; vomiting, 1.1; anorexia, 1.1; hypomagnesemia, 1.1	Acneiform rash, 6; erythema, 5; pruritus, 3; rash, 3; exfoliation, 3; nausea/vomiting, 2; fatigue/asthenia, 2; diarrhea, 2; dyspnea, 1; infections, 6	Rash, 3; fatigue, 3; vomiting, 1; pruritus, 1; nausea, 1; diarrhea, 1; dyspnea, 1
Onset of skin toxicity	12–15 days	NR	6–13 days	NR	9–14 days
Infusion reactions, type, *n* (%)	Infusion reaction, 0 (0); only one grade 2 reaction	Moderate hypersensitivity, 1 (0.6)	Infusion reaction, 1 (1)	Infusion reaction, grade 3 or 4, 7 (3)	Hypersensitivity reaction, 1 (0.7)

**Table III.** Adverse events of trials with combination cetuximab therapy in the treatment of metastatic colorectal cancer^31^

	*BOND (Cunningham 2004) N=212*	*CRYSTAL (Raoul 2009) N=87*	*OPUS (Bokemeyer ECCO 2007) N=337*	*BOND2 (Saltz 2007) N=83*		*CAIRO2 (Tol Ann Oncol 2008) n=192*
Treatment regimen	CET + IRI	CET + IRI +5-FU	CET plus 5-FU/FA/oxaliplatin (FOLFOX-4)	CET + BEV + IRI n=43	CET + BEV N=40	Capecitabine + oxaliplatin + BEV + CET
Any AE	65.1	NR	NR	NR	NR	NR
Skin toxicity	80	52% (rash)	NR	83	85	92
Grade 3/4 AE, %	Anemia 4.7 Neutropenia 9.4 Thrombocytopenia 0.5 Diarrhea 21.2 Asthenia 13.7 Acne-like rash 9.4 Nausea and vomiting 7.1 Abdominal pain 3.3 Stomatitis 2.4 Dyspnea 1.4 Fever 2.4	Leukopenia 21.2 Diarrhea 12 Vomiting 12 Rash 12 Acne 10 Asthenia 10 Intestinal obstruction 10 Abdominal pain 6 Mucous membrane disorder 6 Dyspnea 8 Atrial fibrillation 4 Deep thrombophelbitis 4 Gamma glutamyl transpeptidase increased 4 Hypokalaemia 4 Liver function test abnormal 4 Skin disorder 4 Thrombosis 4 Urinary tract infection 4 Weight loss 4	Neutropenia 27.6 Diarrhea 7.1 Neurotoxicity 3.5 Leukopenia 7.1 Fatigue 3.5 Skin reactions 14.1 Infusion-related reactions 4.1	Skin rash 21 Paronychial cracking 7	Skin rash 20 Paronychial cracking 5 Headache 5	Acneiform skin rash 26 Nail changes 6 Dry skin 0.5 Hand–foot syndrome 16 Diarrhoea 23 Nausea 6 Vomiting 6 Hypertension 4 Proteinuria 0.5 Cardiac ischaemia 2 Cerebrovascular ischaemia 0.5 Sensory neuropathy 6 Thromboembolic event 8 Infection 5 Febrile neutropenia 0 Allergic reaction 7 Bleeding 1 Gastrointestinal perforation 1 Hypomagnesaemia 2

**Table IV.** Grades 3 and 4 events in the STEPP trial^20^

	*Pmab + FOLFIRI Q2W*		*Pmab + Iri Q3W*		*Pmab + FOLFIRI Q2W*		*Pmab + Iri Q3W KRAS*	

	P (n=28)	R (n=27)	P (n=20)	R (n=20)	WT *KRAS* (n=32)	Mut *KRAS* (n=19)	WT *KRAS* (n=17)	Mut *KRAS* (n=19)
Dermatitis acneiform - n (%)	2 (7)	7 (26)	0 (0)	3 (15)	7 (22)	2 (11)	1 (6)	2 (11)
Diarrhea - n (%)	6 (21)	9 (33)	1 (5)	6 (30)	10 (31)	4 (21)	2 (12)	4 (21)
Dehydration - n (%)	3 (11)	7 (26)	0 (0)	6 (30)	5 (16)	4 (21)	1 (6)	4 (21)
Neutropenia - n (%)	3 (11)	7 (26)	1 (5)	5 (25)	5 (16)	3 (16)	3 (18)	3 (16)
Deep vein thrombosis – n (%)	0 (0)	1 (4)	0 (0)	1 (5)	1 (3)	0 (0)	0 (0)	0 (0)

**Table V.** Dose Modifications Guidelines for severe acneiform rash in patients taking cetuximab^78^

*Severe Acneiform Rash*		*Cetuximab*	*Outcome*	*Cetuximab Dose Modification*
1st	occurrence	Delay infusion 1 to 2 weeks	Improvement No improvement	Continue at 250 mg/m^2^ Discontinue cetuximab
2nd	occurrence	Delay infusion 1 to 2 weeks	Improvement No improvement	Reduce dose to 200 mg/m^2^ Discontinue cetuximab
3rd	occurrence	Delay infusion 1 to 2 weeks	Improvement No improvement	Reduce dose to 150 mg/m^2^ Discontinue cetuximab
4th	occurrence	Discontinue ERBITUX		

**Table VI.** Grades of Severity of Hypomagnesemia: National Cancer Institute–Common Toxicity Criteria Version 3

Grade 0	Within normal limits
Grade 1	< LLN–1.2 mg/dL or < LLN–0.5 mmol/L
Grade 2	< 1.2–0.9 mg/dL or < 0.5–0.4 mmol/L
Grade 3	< 0.9–0.7 mg/dL or < 0.4–0.3 mmol/L
Grade 4	< 0.7 mg/dL or < 0.3 mmol/L

Abbreviation: LLN = lower limit of normal
